# Iron Deficiency, Iron Deficiency Anemia, and HbA1c Levels in Nondiabetic Adults: A Systematic Review and Meta-Analysis

**DOI:** 10.7759/cureus.115191

**Published:** 2026-08-25

**Authors:** Mohamed Abdelgadir Hag Elagib, Rowida A Ahmed, Hassan Eltom, Mihad Farah Abdelrahman, Muhsin Mohammed Elhadi Agbna Mohammed, Asim Ahmed, Shougyat Abdelmoniem Mohammed Ahmed

**Affiliations:** 1 Internal Medicine, Dallah Namar Hospital, Riyadh, SAU; 2 General Medicine, NMC Royal Hospital, Abu Dhabi, ARE; 3 Internal Medicine, Omdurman Islamic University, Omdurman, SDN; 4 Faculty of Medicine, University of Khartoum, Khartoum, SDN; 5 Internal Medicine, SouthDoc Company Ltd., Bantry, IRL; 6 Medicine and Surgery, University of Gezira, Wad Madani, SDN; 7 Internal Medicine/Acute Medicine, NMC Royal Hospital, Abu Dhabi, ARE

**Keywords:** anemia, glycated hemoglobin, glycemic misclassification, iron-deficiency, non-diabetic adults

## Abstract

Iron deficiency refers to depleted body iron stores, while iron deficiency anemia (IDA) develops when iron depletion becomes sufficient to impair hemoglobin production. Both conditions may influence glycated hemoglobin (HbA1c) independently of circulating glucose and may therefore affect glycemic classification in adults without diabetes. This systematic review and meta-analysis evaluated the association of iron deficiency and IDA with HbA1c levels, while also examining anemia severity, iron-status markers, the effects of iron replacement, and the potential for HbA1c-based glycemic misclassification. PubMed/MEDLINE and Scopus were searched from inception to the final search, supplemented by Google Scholar and citation tracking. Eligible observational and before-and-after studies included adults without established diabetes and reported HbA1c in relation to iron status. Two reviewers independently performed study screening and data extraction. Risk of bias was assessed using Joanna Briggs Institute tools appropriate to each analytical component. Twenty-five reports were included, representing 24 independent populations. The primary meta-analysis did not establish a conclusive overall difference in HbA1c between participants with IDA and non-anemic controls, while the expanded sensitivity analysis suggested higher HbA1c in IDA but remained affected by substantial heterogeneity and methodological limitations. Individual studies reported inconsistent relationships between HbA1c, anemia severity, and iron-status markers. Most iron-treatment studies reported reductions in HbA1c after treatment, although some reported increases, and incomplete paired-data reporting prevented valid pooling. Evidence also suggested that IDA may contribute to HbA1c-based glycemic misclassification, particularly near diagnostic thresholds. Overall, the available evidence indicates that iron deficiency and IDA may affect HbA1c in adults without diabetes, although the direction and magnitude of this effect remain inconsistent. Accordingly, HbA1c warrants cautious interpretation when iron deficiency or anemia is present, especially when it is discordant with glucose-based testing.

## Introduction and background

Iron deficiency is one of the most common nutritional and hematological disorders worldwide and may occur with or without anemia. Iron deficiency anemia (IDA) develops when depleted iron stores become insufficient to maintain normal erythropoiesis and hemoglobin production. Diagnosis commonly incorporates hemoglobin concentration, ferritin, transferrin saturation, serum iron, and red-cell indices, although the thresholds and combinations of markers used vary across clinical and research settings [1,2]. Beyond its hematological consequences, both iron deficiency and IDA may influence HbA1c interpretation through changes in erythrocyte production, survival, and turnover.

Glycated hemoglobin (HbA1c) is widely used to assess long-term glycemic exposure and to screen for and diagnose prediabetes and diabetes. It reflects the non-enzymatic attachment of glucose to hemoglobin over the preceding approximately eight to 12 weeks and offers practical advantages over fasting plasma glucose and oral glucose tolerance testing because it does not require fasting and is less affected by acute glucose fluctuations [3-5]. Its diagnostic use relies on established HbA1c thresholds, appropriately standardized assays, and confirmatory testing when required [3,4]. However, HbA1c is an indirect measure of glycemia, and its interpretation depends not only on circulating glucose but also on erythrocyte lifespan, hemoglobin characteristics, and assay performance. Analytical or biological interference should therefore be considered when HbA1c values are substantially discordant with glucose-based measurements [3-5].

Alterations in erythrocyte turnover may affect HbA1c independently of glucose concentration. Prolonged erythrocyte survival may increase the time available for hemoglobin glycation and produce higher-than-expected HbA1c values, whereas shortened red-cell survival, hemolysis, acute blood loss, or increased erythropoiesis may produce falsely low values [6]. In iron deficiency, proposed mechanisms include shifts in erythrocyte age distribution, reduced erythropoiesis, altered hemoglobin structure, and increased oxidative stress. Their relative contribution remains uncertain, and the effect on HbA1c may vary with anemia severity, iron-deficiency definitions, assay methods, and study-population characteristics [6,7].

Previous reviews have not established a consistent effect of iron deficiency on HbA1c. A systematic review of anemia and erythrocyte abnormalities suggested that iron deficiency, with or without anemia, generally produced a spurious increase in HbA1c without a corresponding increase in glucose indices, but the evidence was limited and heterogeneous [8]. Another review and meta-analysis reported a positive but statistically inconclusive difference associated with iron deficiency or IDA and combined these conditions with other potential sources of HbA1c interference [9]. More recent reviews have examined HbA1c changes after iron replacement but have often combined diabetic and non-diabetic populations or focused mainly on women, including pregnant populations in whom physiological changes may independently influence iron metabolism and HbA1c interpretation [10,11].

Important uncertainties therefore remain. The available evidence is dominated by small observational studies using heterogeneous definitions of iron deficiency and anemia, different HbA1c assays, variable methods for excluding diabetes, and populations with substantially different anemia severity. Previous reviews have not consistently isolated non-pregnant adults without established diabetes and have often examined baseline differences, treatment effects, or diagnostic misclassification separately rather than integrating these related evidence domains [8-11]. This leaves an important clinical question unresolved: whether altered HbA1c in iron-deficient adults without diabetes reflects a genuine difference in glycemic exposure or interference related to altered hematological status. To address this gap, the present review synthesizes evidence on both iron deficiency and IDA in adults without established diabetes. It considers baseline HbA1c differences, relationships with anemia severity and iron-status markers, changes following iron replacement, and the potential for HbA1c-based glycemic misclassification. The primary objective was to evaluate the association of iron deficiency and IDA with HbA1c levels in adults without diabetes. Secondary objectives were to examine variation according to participant characteristics, exposure definitions, assay methods, study design, and methodological quality.

## Review

Methodology

Review Design and Eligibility

This systematic review and meta-analysis was reported in accordance with the Preferred Reporting Items for Systematic Reviews and Meta-Analyses 2020 statement [12]. An internal protocol was developed before data extraction and quantitative synthesis; however, the review was not prospectively registered.

Baseline association evidence was structured using the Population-Exposure-Comparator-Outcome framework, whereas iron-treatment evidence was structured using the Population-Intervention-Comparator-Outcome framework. The eligibility and synthesis frameworks are summarized in Table 1.

**Table 1:** PECO and PICO frameworks used to define eligibility and synthesis. HbA1c, glycated hemoglobin A1c; IDA, iron deficiency anemia; PECO, Population-Exposure-Comparator-Outcome; PICO, Population-Intervention-Comparator-Outcome

Framework component	Baseline association evidence (PECO)	Iron-treatment evidence (PICO)
Population	Adults aged ≥18 years without established diabetes	Adults aged ≥18 years without established diabetes who had iron deficiency or IDA
Exposure/Intervention	Iron deficiency or IDA	Oral or parenteral iron replacement
Comparator	Iron-replete or non-anemic controls	The same participants before treatment or an untreated comparison group
Outcomes	HbA1c levels, anemia severity, iron-status markers, and glycemic misclassification	Change in HbA1c and hematological measures after treatment
Study designs	Cross-sectional, case-control, cohort, database, and retrospective studies	Controlled or uncontrolled before-and-after studies
Synthesis	Quantitative synthesis when comparable data were available; otherwise narrative synthesis	Narrative synthesis unless valid paired-change data were available

The detailed inclusion and exclusion criteria applied during study selection are presented in Table 2.

**Table 2:** Inclusion and exclusion criteria. Each inclusion and exclusion criterion is presented in a separate row. HbA1c, glycated hemoglobin A1c; IDA, iron deficiency anemia

Criterion	Inclusion	Exclusion
Age	Adults aged ≥18 years	Participants aged <18 years
Diabetes status	No established diabetes mellitus	Established diabetes without separately extractable non-diabetic data
Pregnancy	Non-pregnant adults	Pregnant women
Mixed populations	Eligible adult data separately extractable	Eligible data not separately extractable
Iron status	Iron deficiency or IDA defined by study-specific criteria	Iron deficiency or IDA not clearly defined
Exposure	Iron deficiency or IDA evaluated in relation to HbA1c	Exposure unrelated to iron deficiency or IDA
Intervention	Oral or parenteral iron replacement	Treatment unrelated to iron replacement
Comparator	Non-anemic controls or the same participants before treatment	No eligible comparator or baseline assessment
Outcomes	HbA1c levels, HbA1c change, iron-status associations, or glycemic misclassification	No relevant or extractable HbA1c outcome
Kidney disease	No chronic kidney disease	Chronic kidney disease
Hemolysis	No hemolytic anemia	Hemolytic anemia
Hemoglobin disorders	No major hemoglobinopathy	Major hemoglobinopathy
Malignancy	No malignancy	Malignancy
Blood transfusion	No recent transfusion	Recent blood transfusion
Acute bleeding	No major acute bleeding	Major acute bleeding
Anemia type	Iron deficiency-related anemia	Anemia unrelated to iron deficiency
Study design	Cross-sectional, case-control, cohort, database, chart-review, or before-and-after study	Review, editorial, commentary, protocol, case report, or animal study
Publication status	Peer-reviewed, full-text original study	Preprint, unpublished study, or conference abstract without sufficient data

Iron deficiency without anemia was eligible for narrative synthesis, whereas the conventional meta-analysis was restricted to comparisons between participants with IDA and non-anemic controls. Studies were not excluded according to methodological quality; risk-of-bias findings were used only to interpret the evidence.

PubMed/MEDLINE and Scopus were searched from database inception through July 10, 2026, without publication-date restrictions. Google Scholar was used as a supplementary search source using predefined topic-specific queries and relevance-based ordering, rather than as an exhaustive bibliographic database search. Backward reference-list screening of included studies and relevant reviews and forward citation tracking of key eligible reports were also undertaken during full-text assessment to identify additional potentially eligible studies. Records identified through supplementary searching were incorporated into the overall search log and screened using the same eligibility criteria as database-derived records. No language restriction was applied during the electronic search, and non-English full-text reports were eligible when reliable translation was available. Because Google Scholar and citation tracking were supplementary discovery methods and were not prospectively logged with a fixed screening count, stopping threshold, or source-specific retrieval count, those quantities cannot be reconstructed reliably and are reported as a limitation rather than estimated retrospectively.

Search concepts included iron deficiency, IDA, HbA1c, glycated or glycosylated hemoglobin, non-diabetic populations, iron replacement, and glycemic misclassification. The complete database and supplementary search strategies are presented in Table 3.

**Table 3:** Electronic database and supplementary search strategies. HbA1c, glycated hemoglobin A1c; IDA, iron deficiency anemia; MeSH, Medical Subject Headings; TITLE-ABS-KEY, title, abstract, and keyword search

Source	Search strategy	Coverage and limits	Purpose
PubMed/MEDLINE	("Anemia, Iron-Deficiency"[Mesh] OR "iron deficiency anemia"[Title/Abstract] OR "iron deficiency anaemia"[Title/Abstract] OR "iron-deficiency anemia"[Title/Abstract] OR "iron deficiency"[Title/Abstract] OR IDA[Title/Abstract]) AND ("Glycated Hemoglobin"[Mesh] OR HbA1c[Title/Abstract] OR "hemoglobin A1c"[Title/Abstract] OR "haemoglobin A1c"[Title/Abstract] OR "glycated hemoglobin"[Title/Abstract] OR "glycated haemoglobin"[Title/Abstract] OR "glycosylated hemoglobin"[Title/Abstract])	From database inception to July 10, 2026; no date or language restriction	Main database search
Scopus: Search 1	TITLE-ABS-KEY(("iron deficiency anemia" OR "iron deficiency anaemia" OR "iron-deficiency anemia" OR "iron deficiency" OR IDA) AND (HbA1c OR "hemoglobin A1c" OR "haemoglobin A1c" OR "glycated hemoglobin" OR "glycated haemoglobin" OR "glycosylated hemoglobin"))	From database inception to July 10, 2026; no date restriction	Broad exposure-outcome search
Scopus: Search 2	TITLE-ABS-KEY(("iron deficiency anemia" OR "iron deficiency anaemia" OR "iron deficiency" OR IDA) AND (HbA1c OR "hemoglobin A1c" OR "haemoglobin A1c" OR "glycated hemoglobin" OR "glycosylated hemoglobin") AND (nondiabet* OR "non-diabetic" OR "without diabetes" OR euglyc* OR prediabet* OR misclassif* OR "iron therapy" OR "iron replacement" OR "iron supplementation"))	From database inception to July 10, 2026; no date restriction	Targeted search for non-diabetic, treatment, and classification outcomes
Google Scholar	"iron deficiency anemia" HbA1c non-diabetic; "iron therapy" HbA1c anemia; "glycemic misclassification" iron deficiency	Searched through July 10, 2026, using relevance-based ordering	Supplementary identification of additional reports
Citation tracking	Backward reference-list screening and forward citation searching of included studies and relevant reviews	Conducted during full-text assessment through July 10, 2026	Identification of related or missed studies

Google Scholar, backward reference-list screening, and forward citation tracking were used as supplementary search methods. Records identified through these methods were incorporated into the total number of records identified and documented in the search log. DOI records, publisher webpages, bibliographic information, and full-text reports were used to verify report identity and eligibility but were not treated as separate search sources.

Study Selection and Data Extraction

Retrieved records were managed using a standardized Microsoft Excel workbook. Duplicates were identified using DOI, PubMed identifier, title, author, publication year, journal, and publisher information. Potentially overlapping populations were evaluated using study location, recruitment period, data source, sample size, and participant characteristics. The same participants were not included more than once in a quantitative analysis.

Two reviewers independently screened titles and abstracts and assessed potentially eligible full-text reports. Disagreements were resolved through discussion and consensus. Each excluded full-text report was assigned one primary eligibility-based exclusion reason. A formal inter-reviewer agreement statistic was not calculated.

Two reviewers independently extracted data using a standardized Excel form that was piloted before final extraction. Extracted information included study characteristics, participant and comparator details, definitions of iron deficiency and IDA, HbA1c assay methods, hematological and iron-status markers, baseline and post-treatment outcomes, adjusted estimates, and methodological limitations. Disagreements were resolved through consensus, and study authors were not contacted for missing information.

When numerical values differed between the abstract, text, tables, or figures, the reported analytical population and internal calculations were reviewed. Unresolved discrepancies affecting the mean, standard deviation, sample size, or group definition were excluded from the primary quantitative analysis. Missing values were not imputed unless they could be derived using an established method.

Outcomes

The primary outcome was the raw mean difference in HbA1c percentage points between participants with IDA and non-anemic controls.

Secondary outcomes included associations between HbA1c and iron deficiency without anemia, anemia severity, hemoglobin, ferritin, serum iron, transferrin saturation, and red-cell indices; changes in HbA1c after iron replacement; adjusted population-level associations; and HbA1c-based glycemic misclassification.

Risk-of-Bias Assessment

Risk of bias was assessed independently by two reviewers using tools appropriate to each analytical component. Baseline cross-sectional comparisons, contemporaneous case-control comparisons, population-based associations, severity analyses, correlations, and glycemic-classification outcomes were assessed using the JBI Critical Appraisal Checklist for Analytical Cross-Sectional Studies [13]. Iron-treatment studies were assessed using the JBI Critical Appraisal Checklist for Quasi-Experimental Studies [14].

Each domain was classified as yes, no, unclear, or not applicable. Overall judgments were categorized as low, moderate, or high risk based on exposure and outcome measurement, participant classification, control of confounding, numerical consistency, follow-up, and statistical analysis.

Detailed domain-level judgments were presented in traffic-light plots. Green circles represented low risk or yes, yellow circles represented unclear risk or some concerns, and red circles represented high risk or no. Risk-of-bias findings were used to interpret the evidence and did not determine study eligibility or quantitative inclusion.

Data Synthesis and Statistical Analysis

All included studies were incorporated into a structured narrative synthesis organized by baseline comparisons, anemia severity, iron-status markers, iron-treatment outcomes, glycemic misclassification, and adjusted population-level findings. Differences in population characteristics, exposure definitions, HbA1c assays, study design, baseline glucose, and risk of bias were considered during interpretation.

Studies were eligible for mean-difference pooling when they reported non-overlapping IDA and non-anemic control groups with directly reported sample sizes, mean HbA1c values, and standard deviations. HbA1c was analyzed in percentage points, and positive effects indicated higher HbA1c in the IDA group. Adjusted estimates, correlations, threshold outcomes, and survey-weighted means were synthesized separately.

Random-effects meta-analysis was performed because clinical and methodological heterogeneity was expected. Between-study variance was estimated using restricted maximum likelihood, and modified Hartung-Knapp confidence intervals were calculated [15,16].

The primary model included studies meeting prespecified data-integrity and extractability criteria. An expanded sensitivity analysis included additional studies with usable data but non-critical methodological or reporting concerns. Eligibility for each quantitative model was determined according to whether a study provided suitable, internally consistent data for that specific analysis. Studies could enter the expanded exploratory sensitivity analysis when distinct IDA and non-anemic comparison groups and the required numerical data were separately identifiable. Studies with population overlap or unresolved numerical inconsistencies were not quantitatively pooled.

Heterogeneity was assessed using Cochran’s Q, τ², I², and 95% prediction intervals for both the primary and expanded models. Planned subgroup analyses were not performed because comparable subgroup data were insufficient.

Small-study effects were assessed in the expanded model using funnel-plot inspection and Egger regression [17]. These findings were interpreted cautiously because asymmetry may arise from heterogeneity or methodological differences rather than publication bias alone.

Iron-treatment outcomes were synthesized narratively because most studies did not report the variance of the paired change or the within-participant correlation required for valid pooling. A formal Grading of Recommendations Assessment, Development and Evaluation assessment was not performed; certainty was interpreted narratively according to study design, consistency, precision, directness, risk of bias, and heterogeneity.

Quantitative analyses were implemented using custom Python code with NumPy and SciPy, and figures were generated using Matplotlib. All statistical tests were two-sided, with *P* < 0.05 used as the conventional threshold.

Ethical Considerations

Ethics approval and informed consent were not required because the review used previously published aggregate data and did not involve identifiable individual-level information.

Results

Study Selection

A total of 498 records were identified through database searching and other methods, including one additional report identified during reviewer-requested literature verification. After removal of 149 duplicates, 349 records underwent title and abstract screening, of which 273 were excluded. Seventy-six full-text reports were assessed, and 51 were excluded because of ineligible populations, absence of separately extractable adult or non-diabetic data, inadequate identification of iron deficiency or IDA, absence of a relevant HbA1c outcome, insufficient original or extractable data, or an ineligible study design or publication type.

**Figure 1:**
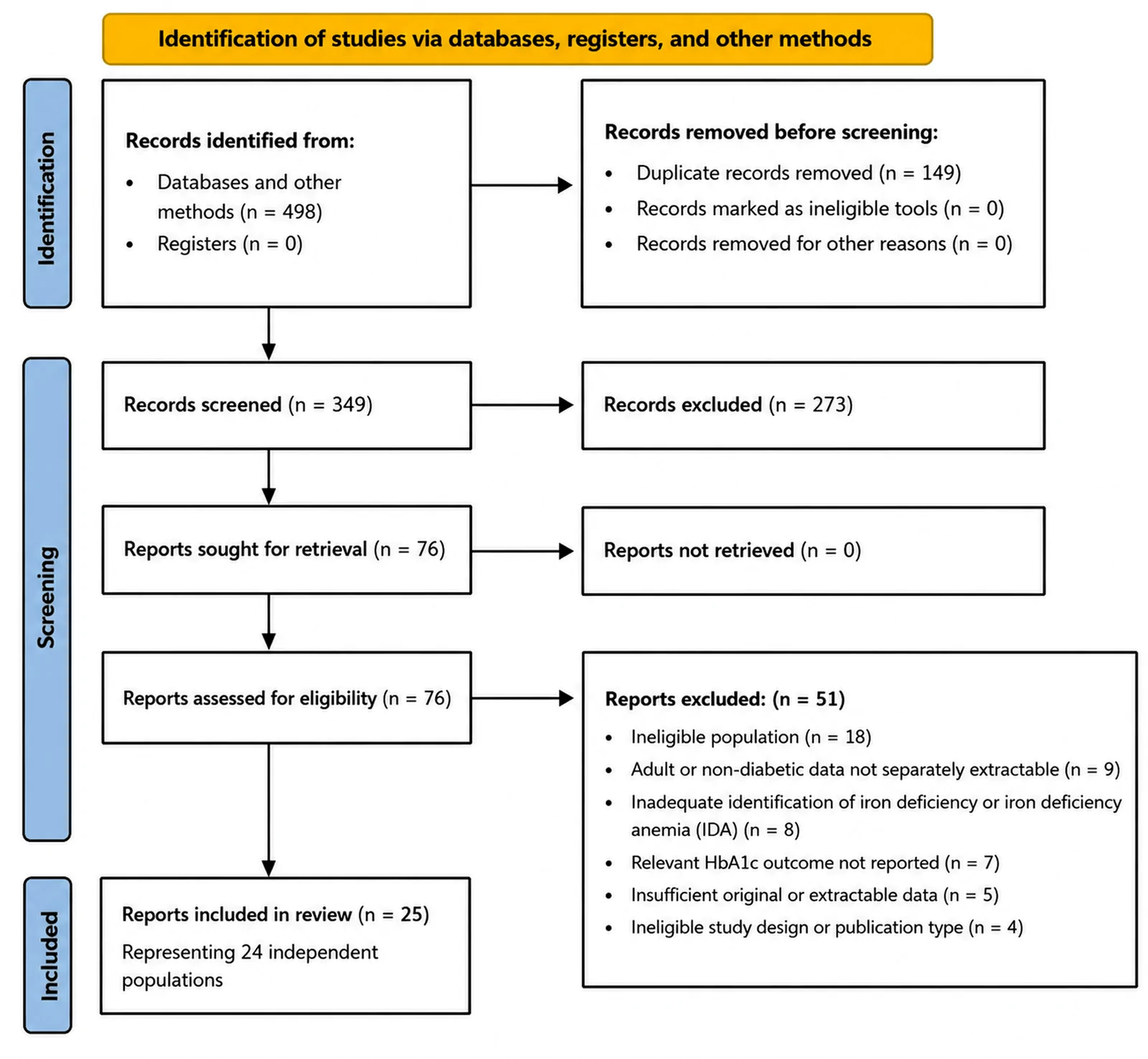
PRISMA 2020 flow diagram of study identification, screening, eligibility assessment, and inclusion. The database and supplementary searches identified 497 records, and one additional report was identified during reviewer-requested literature verification, yielding 498 records in total. After removal of 149 duplicate records, 349 records underwent title and abstract screening, and 273 were excluded. Seventy-six reports were sought and successfully retrieved for full-text assessment. Of these, 51 reports were excluded because of an ineligible population, absence of separately extractable adult or non-diabetic data, inadequate identification of iron deficiency or iron deficiency anemia, absence of a relevant HbA1c outcome, insufficient original or extractable data, or an ineligible study design or publication type. Twenty-five reports were included in the systematic review, representing 24 independent populations. PRISMA, Preferred Reporting Items for Systematic Reviews and Meta-Analyses

Twenty-five reports were included in the qualitative synthesis [18-42], representing 24 independent populations because Kim et al. [18] and Ford et al. [19] reported overlapping National Health and Nutrition Examination Survey populations. Of the 25 included reports, 14 contributed to the expanded exploratory sensitivity analysis, and six met the prespecified criteria for the primary data-integrity-restricted analysis. The study-selection process is presented in Figure 1.

Study Characteristics

The included reports were published between 2010 and 2024. Sixteen were conducted in India, two in the United States, two in Saudi Arabia, two in Egypt, one in South Korea, one in China, and one in Turkey. Most were comparative cross-sectional or contemporaneous case-control studies. Four used population-based datasets, one used prospective cohort follow-up, and nine evaluated HbA1c before and after iron replacement.

Sample sizes ranged from 40 to 10,665 participants. Definitions of iron deficiency and IDA varied across studies. HbA1c was most frequently measured using high-performance liquid chromatography, although capillary electrophoresis, ion-exchange, immunoassay, immunoturbidimetric, and boronate-affinity methods were also used. The principal study characteristics and numerical findings are summarized in Table 4.

**Table 4:** Characteristics and principal numerical findings of the included studies. “-” indicates that the assay method was not reported. Values are mean ± standard deviation unless otherwise stated. Sample values expressed as 50/50 represent IDA/control group sizes. CBC, complete blood count; Hb, hemoglobin; HPLC, high-performance liquid chromatography; ID, iron deficiency; IDA, iron deficiency anemia; MCV, mean corpuscular volume; OGTT, oral glucose tolerance test; RRR, relative risk ratio; sTfR, soluble transferrin receptor; TIBC, total iron-binding capacity; TSAT, transferrin saturation

Study	Country/design; sample	ID/IDA criteria; HbA1c method	Principal HbA1c finding	Synthesis
Kim et al. [18]	United States; population-based; *n* = 10,535	Iron markers; HPLC	Women: 5.31% vs. 5.27%; men: 5.43% vs. 5.29%	Adjusted/threshold
Ford et al. [19]	United States; population-based; *n* = 7,478	Hb + ≥2 iron markers; HPLC	Adjusted coefficient = 0.0558; *P* = 0.065	Adjusted
Hardikar et al. [20]	India; cohort follow-up; *n* = 116	Hb + ferritin; HPLC	Hyperglycemia: 25.9% by HbA1c vs. 10.4% by OGTT	Misclassification
Shanthi et al. [21]	India; case-control; 50/50	Hb + smear + ferritin; immunoinhibition	7.60 ± 0.50% vs. 5.50 ± 0.80%	Primary
Kalasker et al. [22]	India; case-control; 40/40	Hb + indices + smear; ion exchange	5.91 ± 0.47% vs. 6.54 ± 0.39%	Exploratory
Hong et al. [23]	South Korea; population-based; *n* = 10,665	Hb + ferritin/TSAT; HPLC	5.70% vs. 5.59%; HbA1c ≥5.7%: 51.2% vs. 33.9%	Adjusted/threshold
Attard et al. [24]	China; population-based; *n* = 7,308	Ferritin/sTfR + Hb; HPLC	Women with ID: RRR = 0.52; men with IDA: RRR = 2.38	Misclassification
Mulani et al. [25]	India; case-control; 50/50	Hb + smear; immunoassay	9.10 ± 1.08% vs. 5.21 ± 0.45%	Exploratory
Rajagopal et al. [26]	India; cross-sectional; 75/75	Hb + ferritin + iron indices; HPLC	6.84 ± 0.07% vs. 5.12 ± 0.04%	Exploratory
Neki et al. [27]	India; controlled before-after; 50/50	Hb + ferritin + smear; ion exchange	6.12 ± 0.21% → 5.21 ± 0.16%; control 5.24 ± 0.35%	Primary/treatment
Kalairajan et al. [28]	India; controlled before-after; 120/120	Hb + ferritin + indices; capillary electrophoresis	4.62 ± 0.30% → 5.82 ± 0.32%; control 5.45 ± 0.28%	Primary/treatment
Bansal et al. [29]	India; case-control; 100/100	Hb + iron studies; latex assay	6.11% vs. 5.01%; ferritin r = −0.341	Narrative
Purbey et al. [30]	India; before-after; 50/50	Hb + ferritin + smear; -	5.92 ± 0.37% → 5.49 ± 0.42%; control 5.11 ± 0.30%	Exploratory/treatment
Pilla et al. [31]	India; follow-up; *n* = 300	Hb + iron + ferritin; -	Severe: 6.10% → 5.10%; mild/moderate: 5.50% → 4.60%	Severity/treatment
Mohamed et al. [32]	Egypt; before-after; 30/30/30	Hb + ferritin + MCV; -	5.90 ± 0.19% vs. 4.58 ± 0.51%; post-treatment 5.33 ± 0.18%	Exploratory/treatment
Bindayel [33]	Saudi Arabia; women; 21/38	Hb + indices/ferritin; HPLC	5.40 ± 0.40% vs. 5.15 ± 0.36%	Primary
Dhapale et al. [34]	India; single-group before-after; *n* = 150	CBC + ferritin; -	6.66 ± 0.43% → 5.58 ± 0.43% → 4.92 ± 0.36%	Treatment
Aboughazala et al. [35]	Egypt; case-control; 40/35	Hb + ferritin; capillary electrophoresis	5.50 ± 0.50% vs. 4.60 ± 0.50%	Exploratory
D’Sa et al. [36]	India; women with IDA; *n* = 40	Hb + indices + ferritin; immunoassay	Moderate: 5.18 ± 0.35%; severe: 4.50 ± 0.34%	Severity
Chhikara [37]	India; laboratory study; *n* = 1,657	Hb + iron profile	Normal: 5.35% vs. 5.34%; prediabetes: 6.05% vs. 6.02%	Expanded exploratory
Patil et al. [38]	India; IDA cohort; *n* = 65	CBC + ferritin + iron/TIBC; HPLC	Mean 5.67%; 34/65 at 5.7%-6.4%; 7/65 >6.4%	Misclassification
Alzahrani et al. [39]	Saudi Arabia; chart review; 103/104	Hb + MCV + ferritin; HPLC	5.75 ± 0.35% vs. 5.32 ± 0.48%; post-treatment 5.44 ± 0.52%	Primary/treatment
Gharde et al. [40]	India; cross-sectional; 56/56	Hb + laboratory ID findings; immunoturbidimetry	6.04 ± 0.74% vs. 4.91 ± 0.65%	Primary
Patel et al. [41]	India; controlled before-after; *n* = 141 IDA	Hb + ferritin + indices; immunoassay	4.63 ± 0.32% → 5.82 ± 0.34%; change 1.19 ± 0.48%	Treatment
Altuntaş et al. [42]	Turkey; comparative before-after; 131/132	Hb + ferritin; HPLC	5.40 ± 0.50% vs. 5.90 ± 0.50%; post-treatment 5.50 ± 0.30%	Expanded exploratory/treatment

HbA1c Levels in Iron Deficiency and IDA

Across settings, the direction of the association was not uniform. Most hospital-based comparative studies reported higher HbA1c in participants with IDA than in non-anemic controls [21,25,26,29,32,35,39,40], and several reported comparable fasting or postprandial glucose levels between groups.

Population-based studies generally showed smaller differences, with modestly higher HbA1c in some analyses [18,23] and a statistically inconclusive adjusted association in another [19].

Conversely, some studies reported lower HbA1c in IDA [22,28,42], nearly identical values [37], or a small statistically inconclusive difference [33], reinforcing the inconsistency in direction across study settings.

Anemia Severity and Iron-Status Markers

Rajagopal et al. [26] reported mean HbA1c values of 5.16%, 6.57%, 7.19%, and 8.01% among non-anemic, mild, moderate, and severe IDA groups, respectively. Gharde et al. [40] reported HbA1c values of 6.6%-7.5% in 7.1% of participants with moderate IDA and 42.8% with severe IDA. Pilla et al. [31] also reported higher baseline HbA1c in severe IDA than in mild-to-moderate IDA.

In contrast, Bindayel [33] reported similar values in mild and moderate-to-severe IDA, D’Sa et al. [36] reported lower HbA1c in severe than in moderate IDA, and Alzahrani et al. [39] reported 5.81% versus 5.69% in lower- and higher-hemoglobin groups.

Correlations were also inconsistent. Bansal et al. [29], Bindayel [33], and Patil et al. [38] reported inverse associations between HbA1c and hemoglobin or iron-status markers. Kalasker et al. [22] and D’Sa et al. [36] reported positive associations, while Chhikara [37] reported no significant relationship.

Changes Following Iron Replacement

Nine studies assessed HbA1c after iron replacement. Reductions were reported by Neki et al. [27], Purbey et al. [30], Pilla et al. [31], Mohamed et al. [32], Dhapale et al. [34], and Alzahrani et al. [39]. Reported decreases ranged from approximately 0.31 to 1.74 percentage points.

In contrast, Kalairajan et al. [28] reported an increase from 4.62% to 5.82%, Altuntaş et al. [42] reported a smaller increase from 5.40% to 5.50% after iron treatment (p = 0.057), and Patel et al. [41] reported an increase from 4.63% to 5.82%, with a mean paired change of 1.19 percentage points. In Patel et al. [41], the numerical tables showed an increase, whereas the narrative conclusion described a decrease.

Treatment outcomes were not pooled because most studies did not report the standard deviation of the paired change or the baseline-to-follow-up correlation. Only Patel et al. [41] reported a directly usable paired-change variance.

HbA1c-Based Glycemic Misclassification

Hardikar et al. [20] reported hyperglycemia in 25.9% of participants using HbA1c compared with 10.4% using OGTT. Only 20% of HbA1c-defined prediabetes or diabetes was confirmed by OGTT, decreasing to 7% among participants with anemia.

Hong et al. [23] reported HbA1c values of at least 5.7% in 51.2% of participants with IDA compared with 33.9% of participants without anemia, without a significant difference at the 6.5% threshold. In a separate population-based analysis, Kim et al. [18] reported stronger associations at lower HbA1c thresholds.

Attard et al. [24] reported sex-specific discordance between fasting glucose and HbA1c. Patil et al. [38] found HbA1c values of 5.7%-6.4% in 34 of 65 normoglycemic participants and values above 6.4% in seven participants, illustrating potential HbA1c-based misclassification despite normoglycemia. In the treatment setting, Purbey et al. [30] reported a decrease in HbA1c-defined prediabetes from 80% before treatment to 30% after treatment, showing that classification also changed after iron replacement.

*Meta-Analysis* 

Eligibility for quantitative synthesis: Fourteen studies provided extractable data for quantitative synthesis. Six met the prespecified data-integrity and extractability criteria for the primary meta-analysis; the remaining eight contributed only to the expanded exploratory sensitivity analysis.

The expanded exploratory sensitivity analysis included the six primary studies and eight additional studies for which an identifiable mean difference could be calculated despite important methodological or reporting concerns. These concerns included incomplete biochemical confirmation of iron deficiency, limitations in group definition or comparability, incomplete reporting of potential confounders, and other methodological issues that reduced confidence in the estimates but did not prevent their calculation.

Model membership was therefore determined by the integrity and extractability of the numerical data rather than by the overall risk-of-bias judgment. Positive mean differences indicated higher HbA1c levels among participants with IDA than among non-anemic controls.

Primary data-integrity-restricted analysis: The primary random-effects meta-analysis included six studies comprising 400 participants with IDA and 418 non-anemic controls. The pooled raw mean difference in HbA1c was 0.66 percentage points (95% confidence interval (CI), -0.37 to 1.68), which was not statistically significant.

Between-study heterogeneity was extreme (Cochran’s *Q*(5) = 1104.74, *P* < 0.001; *I*² = 99.5%; *τ*² = 0.947), and the 95% prediction interval ranged from -2.26 to 3.58 percentage points. This interval indicates that setting-specific effects could plausibly range from lower to substantially higher HbA1c in participants with IDA.

Because both the confidence interval and prediction interval crossed the null value and heterogeneity was extreme, the primary analysis did not support a consistent direction of association across settings. The pooled mean difference should therefore be interpreted cautiously rather than as a stable overall effect.

**Figure 2:**
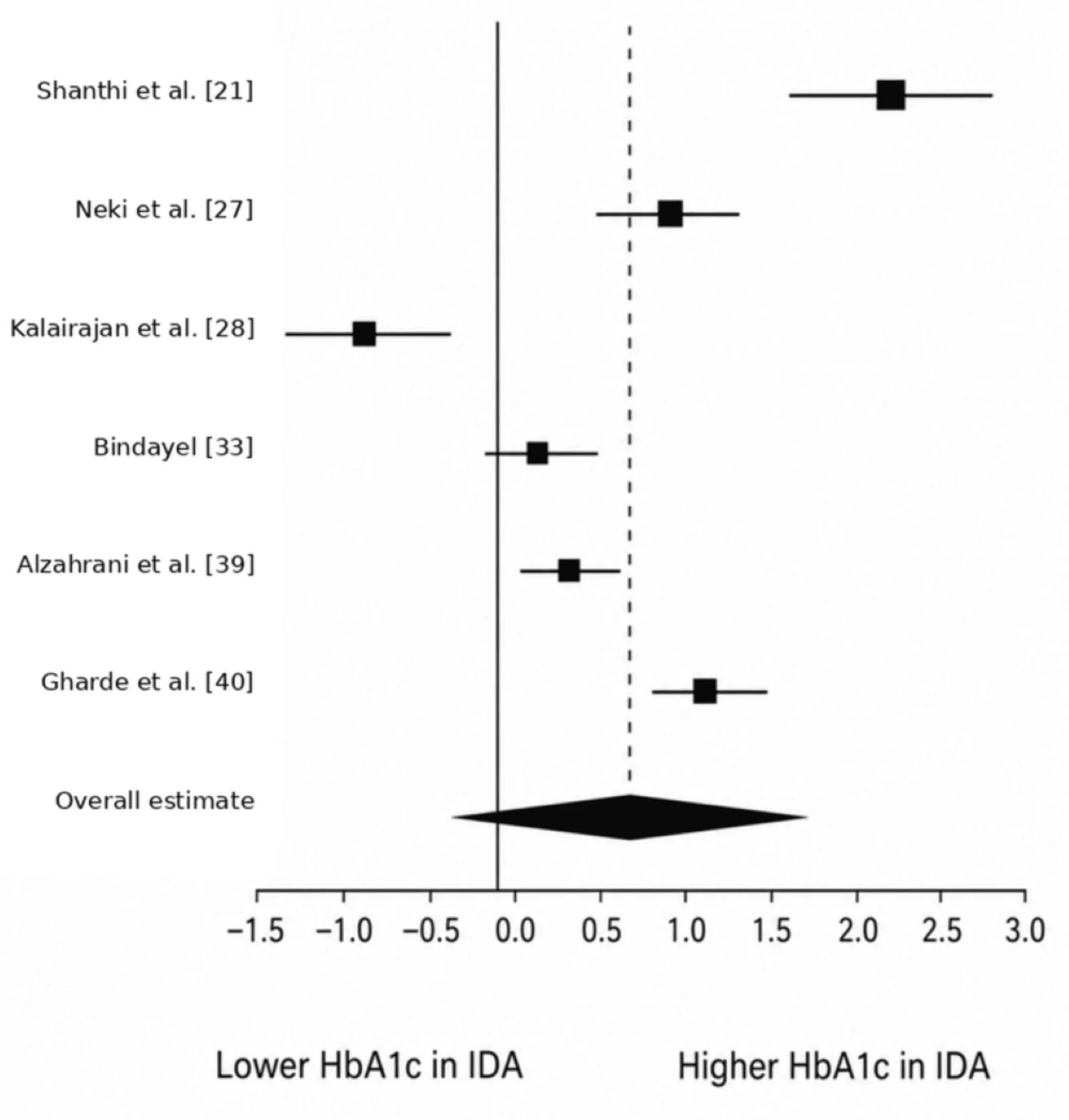
Forest plot of the data-integrity-restricted meta-analysis. Study-specific and pooled mean differences are expressed in HbA1c percentage points with 95% confidence intervals. Positive mean differences indicate higher HbA1c in participants with IDA than in non-anemic controls. The solid vertical line represents the null effect at zero, and the dashed vertical line represents the pooled mean difference. IDA, iron deficiency anemia; HbA1c, glycated hemoglobin

The primary data-integrity-restricted meta-analysis is presented in Figure 2.

Expanded exploratory sensitivity analysis: The expanded exploratory random-effects meta-analysis included 14 studies comprising 1,208 participants with IDA and 1,461 non-anemic controls. The pooled raw mean difference was 0.82 percentage points (95% CI, 0.10-1.53), representing a statistically significant higher average HbA1c level among participants with IDA.

Heterogeneity remained extreme (Cochran’s *Q*(13) = 12,850.67, *P* < 0.001; *I*² = 99.9%; *τ*² = 1.509), and the 95% prediction interval ranged from -1.96 to 3.59 percentage points and crossed the null value. Thus, despite the statistically significant pooled average, the direction and magnitude of the association remained highly inconsistent across studies.

The expanded estimate should consequently be regarded as exploratory rather than as evidence of a uniform increase in HbA1c among all adults with IDA. Differences in anemia severity, iron-deficiency definitions, participant characteristics, comparator selection, HbA1c assay methods, baseline glycemic status, and control of confounding probably contributed to the observed heterogeneity.

**Figure 3:**
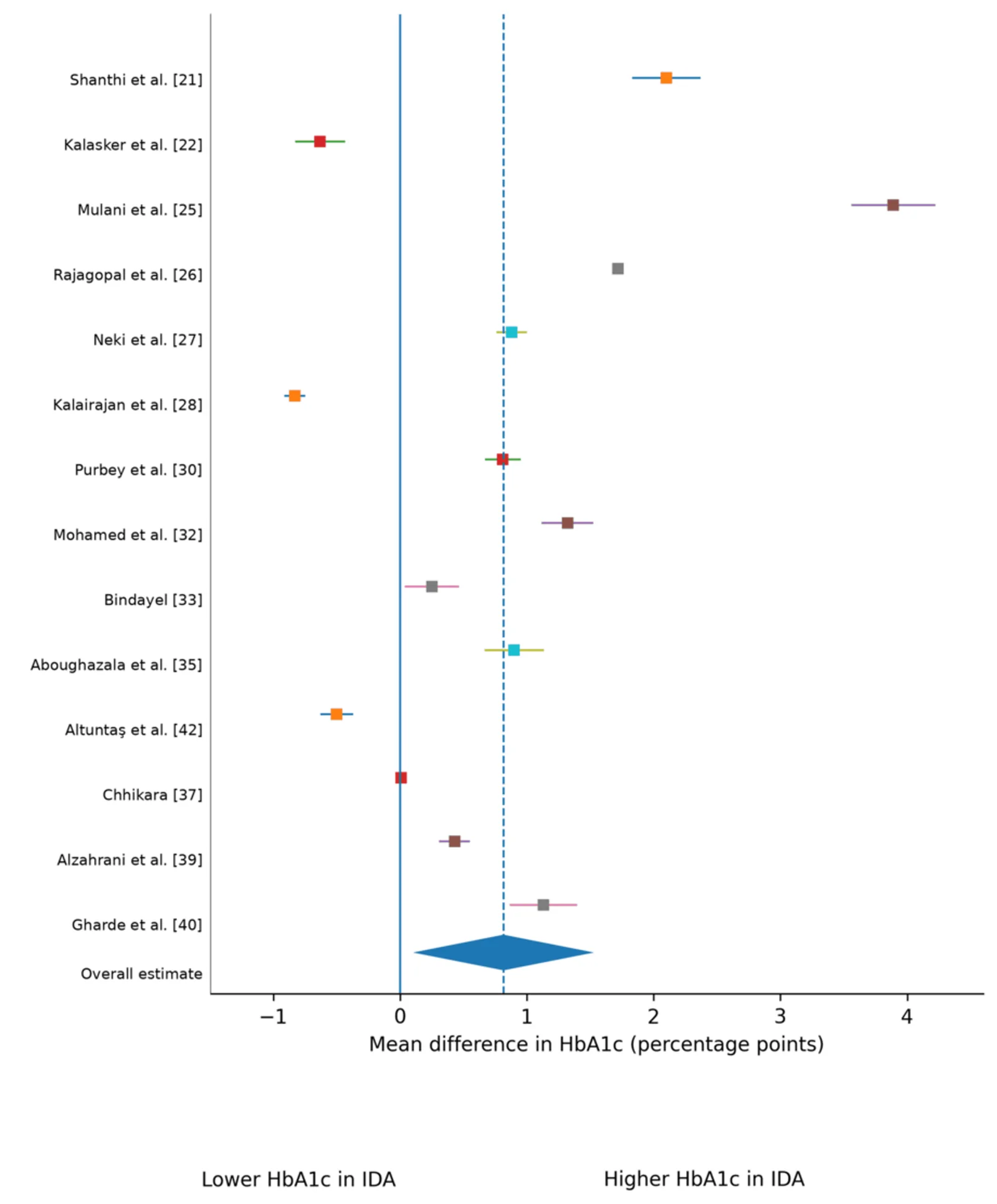
Forest plot of the expanded exploratory meta-analysis of HbA1c levels in iron deficiency anemia. The figure presents the expanded random-effects meta-analysis of 14 studies comparing mean HbA1c levels between adults with iron deficiency anemia and non-anemic controls. Squares represent individual study mean differences, horizontal lines represent 95% confidence intervals, and the diamond represents the pooled effect estimate. The solid vertical line indicates no difference between groups, while the dashed vertical line indicates the pooled mean difference. The pooled mean difference was 0.82 percentage points (95% CI, 0.10-1.53), with extreme heterogeneity (*I*² = 99.9%) and a 95% prediction interval of -1.96 to 3.59. This expanded model was considered exploratory because it included studies with important methodological or reporting concerns that did not prevent effect-size calculation.

The expanded exploratory meta-analysis is presented in Figure 3.

Comparison of the Primary and Expanded Models

Both models showed extreme heterogeneity and wide prediction intervals spanning negative, null, and positive effects, indicating substantial clinical uncertainty. Against this background, the primary model had a confidence interval crossing the null value, whereas the expanded model produced a statistically significant pooled average estimate.

A single pooled mean difference therefore does not adequately represent the variability among the included populations and study methods. The primary model provides the estimate based on the stricter prespecified data-integrity criteria, whereas the expanded model shows how the pooled result changes when additional studies with numerically usable data and additional methodological or reporting concerns are included.

The results of the primary and expanded random-effects meta-analyses, together with the studies included in each model, are summarized in Table 5.

**Table 5:** Summary of the primary and expanded random-effects meta-analyses. Positive mean differences indicate higher HbA1c levels among participants with IDA than among non-anemic controls. The primary model applied stricter data-integrity and extractability criteria. The expanded exploratory model included additional studies with calculable effect estimates but important methodological or reporting concerns. CI, confidence interval; HbA1c, glycated hemoglobin A1c; IDA, iron deficiency anemia

Analysis	Included studies	Studies, n	IDA/control participants	Mean difference, percentage points (95% CI)	*I*²	95% prediction interval
Primary data-integrity-restricted analysis	[21,27,28,33,39,40]	6	400/418	0.66 (−0.37 to 1.68)	99.5%	-2.26 to 3.58
Expanded exploratory sensitivity analysis	[21,22,25-28,30,32-33,35,37,39-40]	14	1,208/1,461	0.82 (0.10 to 1.53)	99.9%	-1.96 to 3.59

Small-Study Effects 

Small-study effects were examined in the expanded 14-study model using visual inspection of the funnel plot and Egger’s regression test. The funnel plot showed variability in the distribution of study estimates and some visual asymmetry. Both assessments were considered exploratory because interpretation is limited by the extreme clinical and statistical heterogeneity among the included studies.

Egger’s regression test did not provide statistically significant evidence of small-study effects (*P* = 0.210). This non-significant result should not be interpreted as evidence of the absence of small-study effects or publication bias, particularly in the presence of extreme heterogeneity and a relatively small number of included studies.

**Figure 4:**
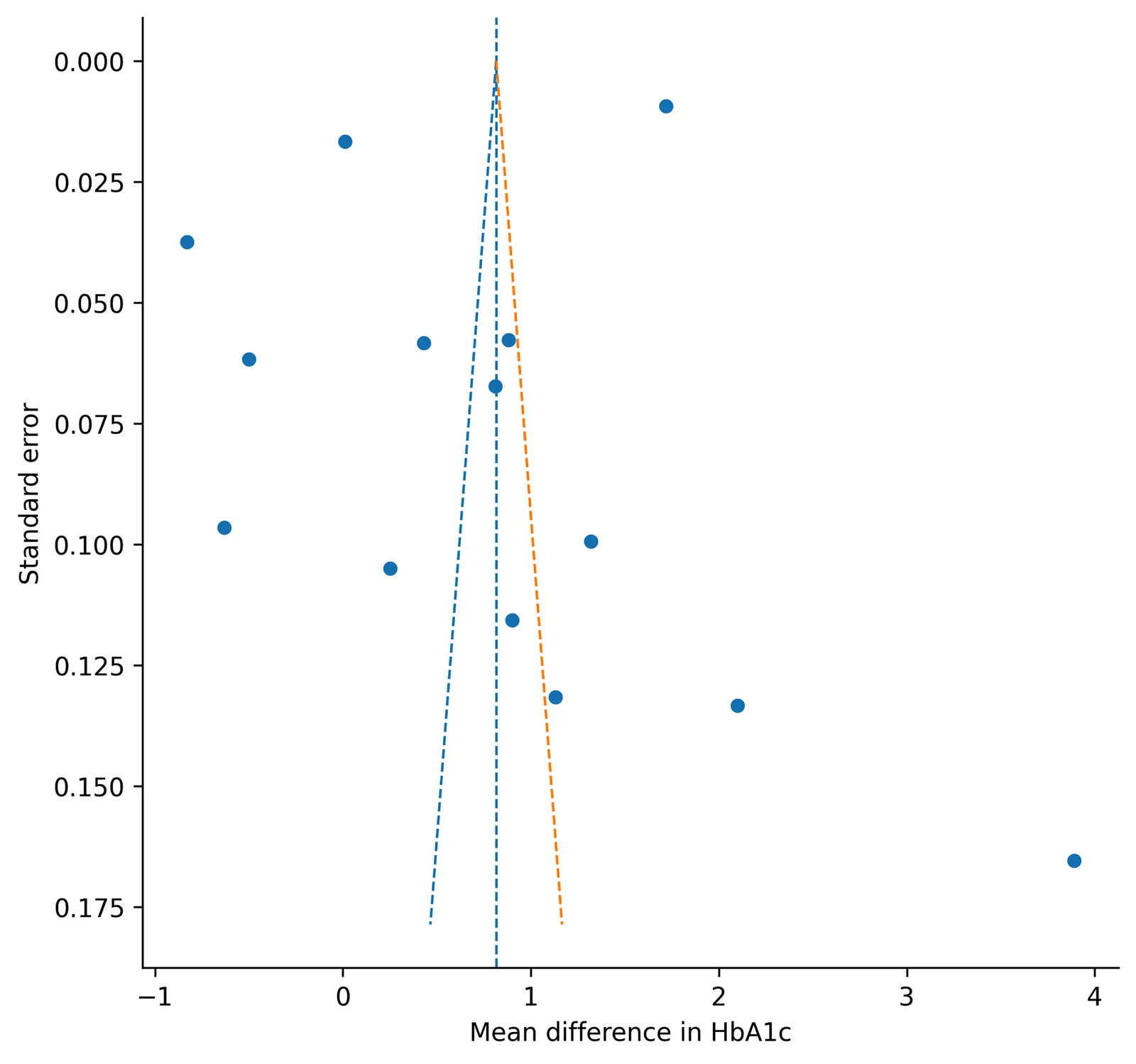
Funnel plot of the expanded meta-analysis assessing small-study effects. The funnel plot displays the mean difference in HbA1c between adults with iron deficiency anemia and non-anemic controls against the corresponding standard error for the 14 studies included in the expanded meta-analysis. The vertical dashed line represents the pooled mean difference of 0.82 percentage points, and the diagonal dashed lines represent the expected pseudo-95% confidence limits around the pooled estimate. Visual assessment of funnel-plot asymmetry and Egger’s regression was considered exploratory because of the extreme between-study heterogeneity. Egger’s regression yielded *P* = 0.210; however, this non-significant result should not be interpreted as evidence of the absence of small-study effects or publication bias.

The funnel plot for the expanded exploratory analysis is presented in Figure 4.

Risk of Bias

Baseline and association evidence: Twenty-four studies contributed baseline, association, severity, correlation, or diagnostic-classification evidence. Five studies were judged to have low overall risk of bias, eight had moderate risk, and 11 had high risk.

The low-risk evidence was mainly derived from large population-based surveys or well-characterized cohort analyses that used objective iron-status definitions, standardized HbA1c assays, and multivariable statistical analyses.

The most frequent concerns among moderate- and high-risk studies were inadequate control of confounding, incomplete biochemical confirmation of iron deficiency, unclear HbA1c assay standardization, and weaknesses in comparator selection or numerical consistency.

**Figure 5:**
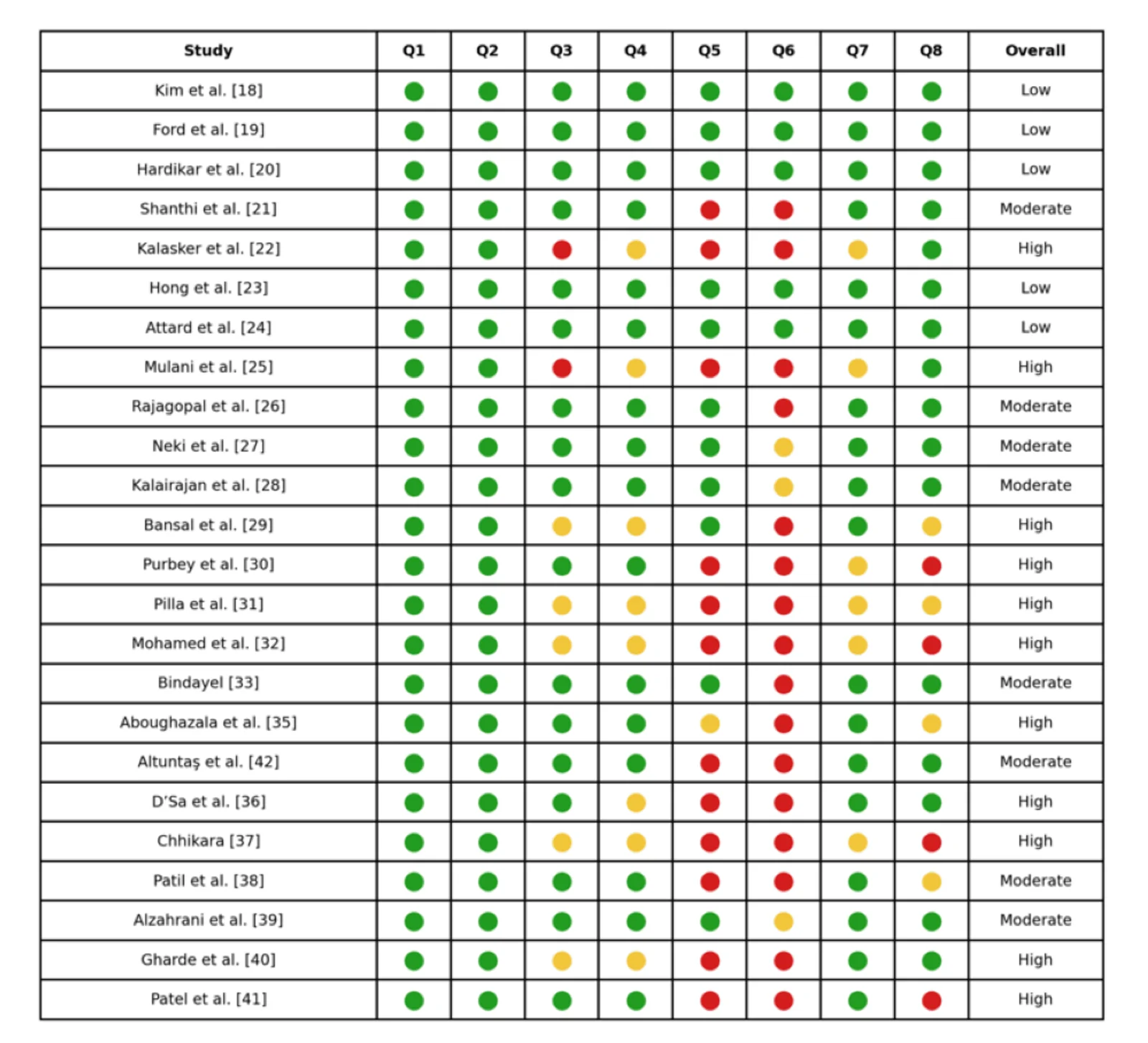
Domain-level risk-of-bias assessment of baseline and observational studies. Domain definitions: Q1, clear eligibility criteria; Q2, participants and study setting adequately described; Q3, iron deficiency or iron deficiency anemia measured validly; Q4, objective diagnostic criteria used; Q5, important confounding factors identified; Q6, strategies used to address confounding; Q7, HbA1c measured validly and reliably; Q8, appropriate statistical analysis used. Color key: 🟢, yes or low concern; 🟡, unclear or insufficient reporting; 🔴, no or high concern. HbA1c, glycated hemoglobin A1c; IDA, iron deficiency anemia; JBI, Joanna Briggs Institute

The domain-level risk-of-bias judgments for baseline and association studies are presented in Figure 5.

Iron-treatment evidence: Nine studies contributed before-and-after iron-treatment evidence. None was judged to have low overall risk of bias. Four studies had moderate risk of bias, and five had high risk.

Methodological limitations mainly involved the absence of multiple pre-intervention measurements and differences in treatment route or dosage according to anemia severity. Reporting limitations included incomplete follow-up and attrition reporting, unavailable paired-change variances, incomplete treatment details, and contradictions between numerical tables and narrative conclusions.

**Figure 6:**
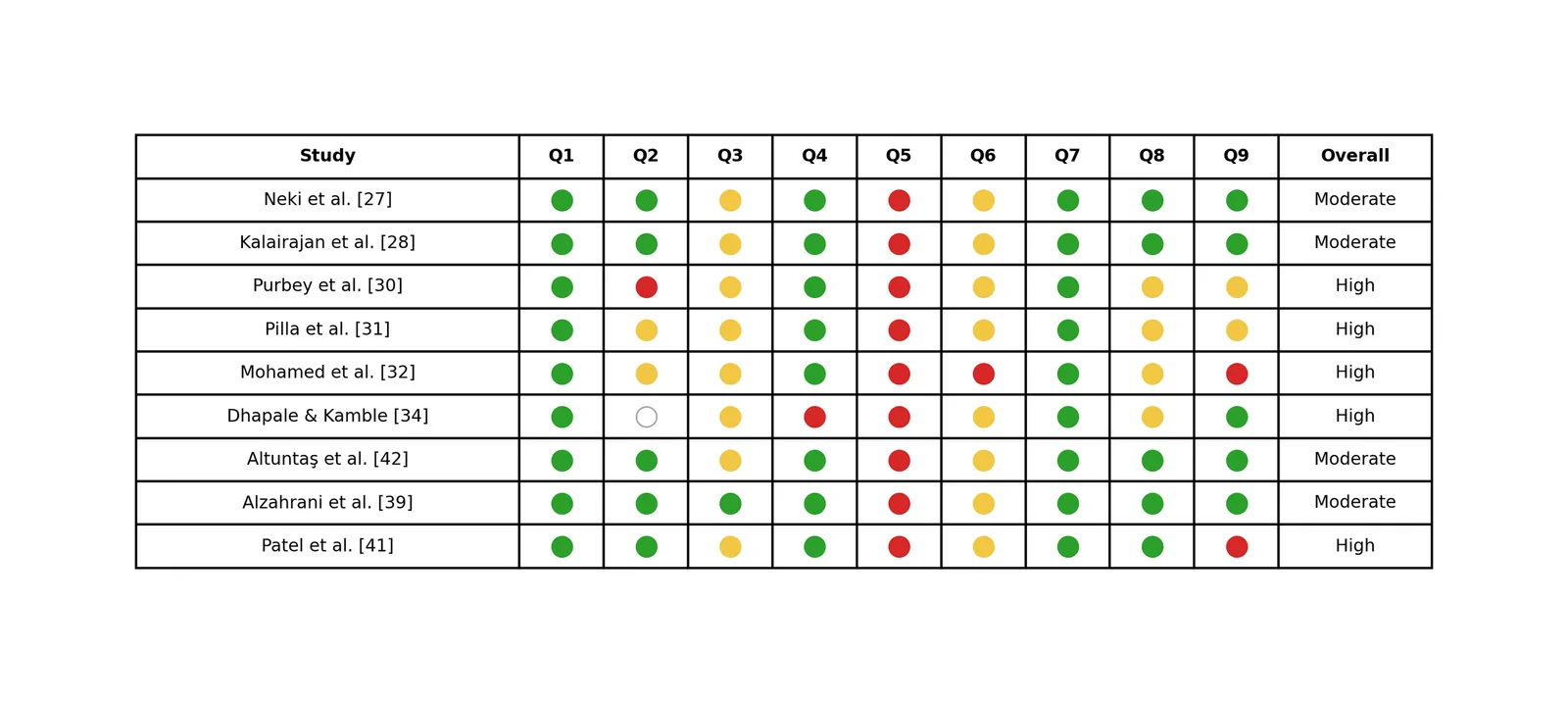
Domain-level risk-of-bias assessment of iron-treatment studies. Domain definitions: Q1, intervention clearly preceded the outcome; Q2, comparison participants were similar; Q3, participants received similar care apart from iron therapy; Q4, a concurrent control group was included; Q5, multiple outcome measurements were obtained before and after treatment; Q6, follow-up was complete or adequately described; Q7, outcomes were measured consistently across groups or time points; Q8, outcome measurement was reliable; Q9, appropriate statistical analysis was used. Color key: 🟢, yes or low concern; 🟡, unclear or insufficient reporting; 🔴, no or high concern; ⚪, not applicable. HbA1c, glycated hemoglobin A1c; JBI, Joanna Briggs Institute

The domain-level risk-of-bias judgments for iron-treatment studies are presented in Figure 6. 

Overall Risk-of-Bias Interpretation

The six studies included in the primary data-integrity-restricted meta-analysis did not represent a uniformly low-risk evidence set. Five of these studies were judged to have moderate risk of bias, while Gharde et al. [40] was judged to have high risk.

The large population-based studies with low risk of bias could not be incorporated into the conventional raw mean-difference meta-analysis because they reported adjusted survey estimates, standard errors, relative risk ratios, or diagnostic-classification outcomes rather than directly usable group means and standard deviations.

Therefore, the primary model should be described as a data-integrity-restricted analysis rather than as a low-risk-of-bias analysis. Risk of bias, extreme statistical heterogeneity, differences in exposure definitions, and variation in comparator selection should all be considered when interpreting the pooled estimates.

The domain definitions and judgments above correspond to the RoB_Methods, RoB_Observational, and RoB_Treatment sheets in the analysis workbook.

Discussion

Principal Findings

This review did not identify a uniform effect of iron deficiency or IDA on HbA1c in adults without established diabetes. The primary data-integrity-restricted analysis did not show a conclusive overall difference, whereas the expanded exploratory analysis suggested higher HbA1c when additional numerically usable studies were included. However, the extreme heterogeneity and wide prediction intervals in both models indicate that the direction and magnitude of the association vary substantially across settings. A single pooled estimate therefore did not adequately represent the variation across populations and study methods.

Across individual studies, the direction of the association was inconsistent. Hospital-based studies more often reported higher HbA1c in participants with IDA, whereas population-based studies generally showed smaller or statistically inconclusive differences after accounting for population characteristics and glycemic status [18,19,21,23,25,26,32,35,40]. Other studies reported lower or nearly unchanged HbA1c values [22,28,37,42]. Taken together, these findings suggest that the observed association is highly dependent on study population, clinical setting, and methodological characteristics rather than reflecting a uniform biological effect.

These findings partly agree with the previous systematic review by English et al. [8], which suggested that iron deficiency may spuriously increase HbA1c but emphasized the limited and heterogeneous evidence. The present review extends that evidence by showing that the main clinical concern is not simply whether iron deficiency raises or lowers HbA1c, but whether altered iron status reduces confidence in HbA1c interpretation when values are close to diagnostic thresholds or are discordant with glucose-based measurements.

Anemia Severity and Iron-Status Markers

Evidence for a severity-dependent association was inconsistent. Some studies reported higher HbA1c with increasing anemia severity [26,31,40], whereas others found similar, lower, or only minimally different values across severity groups [33,36,39]. Associations with hematological and iron-status markers were also inconsistent, with inverse relationships reported in some studies [29,33,38] and positive or null relationships in others [22,36,37]. Overall, the available evidence does not support a reliable dose-response relationship between the severity of iron deficiency or anemia and the direction or magnitude of HbA1c change.

Effects of Iron Replacement

Iron-treatment studies also produced conflicting results. Most reported reductions in HbA1c after iron replacement [27,30-32,34,39], whereas increases were reported in three studies [28,41,42]. Patel et al. [41] also contained a contradiction between the numerical results, which showed an increase, and the narrative conclusion, which described a decrease.

Interpretation of the treatment evidence was limited by heterogeneous treatment regimens, variable follow-up periods, incomplete reporting of paired sample sizes, and the absence of paired-change variances in most studies. These limitations prevented a valid pooled treatment estimate.

A previous review combining diabetic and non-diabetic populations found that most studies reported lower HbA1c after iron replacement [10]. In contrast, the present review suggests that this pattern should not be assumed in non-diabetic adults because the direction of change may depend on anemia severity, treatment response, and assay-related factors.

Potential for HbA1c-Based Glycemic Misclassification

Several studies suggested that iron deficiency or IDA may influence HbA1c-based classification, particularly near the prediabetes threshold. Hardikar et al. [20] reported substantially more participants classified as hyperglycemic using HbA1c than using oral glucose tolerance testing, with particularly poor agreement among participants with anemia. Hong et al. [23] reported a higher frequency of HbA1c values within the prediabetes range among participants with IDA, although the difference was not clearly maintained at the diabetes threshold.

Kim et al. [18] also found a stronger association at lower HbA1c thresholds. Attard et al. [24] reported sex-specific and directionally mixed discordance between HbA1c and fasting glucose, while Patil et al. [38] identified prediabetes- or diabetes-range HbA1c values in several normoglycemic participants with IDA. Purbey et al. [30] reported a reduction in HbA1c-defined prediabetes following iron treatment.

This may be the most clinically important implication of the review. The concern is less about a consistent upward or downward shift in HbA1c and more about whether iron deficiency or IDA can alter classification when values lie near diagnostic thresholds. When HbA1c is discordant with fasting glucose, oral glucose tolerance testing, or the clinical presentation, iron and hematological status should be considered before confirming glycemic classification.

Sources of Heterogeneity and Contribution of the Review

Clinical sources of heterogeneity included differences in iron-deficiency definitions, anemia severity, baseline glycemia, participant sex, and clinical setting. Hospital-based studies often included smaller samples and more severe anemia, whereas population-based studies included broader and generally less severe exposure distributions. Studies relying mainly on red-cell morphology may also have included causes of microcytic anemia other than iron deficiency.

Analytical and methodological sources included variation in HbA1c assay methods, assay standardization, exposure definitions, comparator selection, and control of confounding. The included studies used high-performance liquid chromatography, ion-exchange methods, immunoassays, immunoturbidimetric assays, and capillary electrophoresis, while several did not report the assay method or standardization.

Smaller clinical studies often reported larger effects than nationally representative analyses, but this should not be interpreted as an effect of sample size alone. Rather, smaller studies may also differ in case severity, participant selection, study design, and other methodological characteristics that influence the observed association.

This review specifically focused on adults without established diabetes and integrated baseline HbA1c differences, anemia severity, iron-status markers, response to iron replacement, and glycemic misclassification. It also distinguished a primary analysis based on stricter prespecified data-integrity criteria from an expanded exploratory analysis that examined how the pooled result changed when additional numerically usable studies were included.

Strengths and Limitations

The strengths of this review included predefined eligibility criteria, independent study selection and data extraction, design-appropriate risk-of-bias tools, and separate narrative and quantitative syntheses. Random-effects models, modified Hartung-Knapp confidence intervals, and prediction intervals allowed more cautious interpretation than reliance on the pooled estimate alone.

Several limitations should be considered. The review was not prospectively registered, although an internal protocol was developed before final extraction and analysis. The principal search was limited to PubMed/MEDLINE and Scopus, supplemented by Google Scholar and citation tracking; therefore, studies indexed exclusively in other databases may have been missed. The supplementary Google Scholar and citation-tracking searches were not prospectively logged with fixed screening counts or stopping thresholds, and these quantities therefore cannot be reconstructed reliably after completion of the search. The final search date was July 10, 2026.

The evidence was predominantly observational, and many studies had moderate or high risk of bias because of inadequate confounding control, incomplete biochemical confirmation of iron deficiency, small or imbalanced samples, unclear assay standardization, and internal reporting inconsistencies. Heterogeneity was extreme in both quantitative models, and the prediction intervals crossed the null, limiting the clinical applicability of the pooled estimates.

Treatment evidence was also limited by incomplete paired-change reporting, preventing a valid pooled treatment analysis. Overlapping National Health and Nutrition Examination Survey reports required careful handling, and study authors were not contacted for missing information. A formal Grading of Recommendations Assessment, Development and Evaluation assessment was not performed, and publication bias could not be excluded.

Clinically, the findings suggest that HbA1c should not be interpreted in isolation when iron deficiency or IDA is present. Particular caution is warranted when HbA1c lies near a diagnostic threshold or conflicts with fasting glucose, oral glucose tolerance testing, or the broader clinical picture.

## Conclusions

Iron deficiency and IDA may alter HbA1c levels in non-diabetic adults, but the direction and magnitude of this effect remain inconsistent. The available evidence also suggests that IDA may affect HbA1c-based classification, particularly near the prediabetes threshold, while the effect of iron replacement remains uncertain.

HbA1c should therefore be interpreted cautiously when clinically significant iron deficiency or anemia is present, especially when the result is discordant with fasting glucose, oral glucose tolerance testing, or the patient’s clinical profile. Future studies should use standardized definitions of iron deficiency, validated HbA1c assays, appropriately matched comparison groups, adequate adjustment for major confounders, and complete reporting of treatment outcomes.
